# Brain Metabolic Changes in Rats following Acoustic Trauma

**DOI:** 10.3389/fnins.2017.00148

**Published:** 2017-03-24

**Authors:** Jun He, Yejin Zhu, Jiye Aa, Paul F. Smith, Dirk De Ridder, Guangji Wang, Yiwen Zheng

**Affiliations:** ^1^Key Laboratory of Drug Metabolism and Pharmacokinetics, China Pharmaceutical UniversityNanjing, Jiangsu, China; ^2^Department of Pharmacology and Toxicology, School of Biomedical Sciences, University of OtagoDunedin, New Zealand; ^3^Brain Health Research Centre, University of OtagoDunedin, New Zealand; ^4^Brain Research New ZealandDunedin, New Zealand; ^5^Eisdell Moore Centre for Hearing and Balance Research, University of AucklandAuckland, New Zealand; ^6^Department of Neurosurgery, Dunedin Medical School, University of OtagoOtago, New Zealand

**Keywords:** metabolomics, acoustic trauma, tinnitus, brain, rats

## Abstract

Acoustic trauma is the most common cause of hearing loss and tinnitus in humans. However, the impact of acoustic trauma on system biology is not fully understood. It has been increasingly recognized that tinnitus caused by acoustic trauma is unlikely to be generated by a single pathological source, but rather a complex network of changes involving not only the auditory system but also systems related to memory, emotion and stress. One obvious and significant gap in tinnitus research is a lack of biomarkers that reflect the consequences of this interactive “tinnitus-causing” network. In this study, we made the first attempt to analyse brain metabolic changes in rats following acoustic trauma using metabolomics, as a pilot study prior to directly linking metabolic changes to tinnitus. Metabolites in 12 different brain regions collected from either sham or acoustic trauma animals were profiled using a gas chromatography mass spectrometry (GC/MS)-based metabolomics platform. After deconvolution of mass spectra and identification of the molecules, the metabolomic data were processed using multivariate statistical analysis. Principal component analysis showed that metabolic patterns varied among different brain regions; however, brain regions with similar functions had a similar metabolite composition. Acoustic trauma did not change the metabolite clusters in these regions. When analyzed within each brain region using the orthogonal projection to latent structures discriminant analysis sub-model, 17 molecules showed distinct separation between control and acoustic trauma groups in the auditory cortex, inferior colliculus, superior colliculus, vestibular nucleus complex (VNC), and cerebellum. Further metabolic pathway impact analysis and the enrichment overview with network analysis suggested the primary involvement of amino acid metabolism, including the alanine, aspartate and glutamate metabolic pathways, the arginine and proline metabolic pathways and the purine metabolic pathway. Our results provide the first metabolomics evidence that acoustic trauma can induce changes in multiple metabolic pathways. This pilot study also suggests that the metabolomic approach has the potential to identify acoustic trauma-specific metabolic shifts in future studies where metabolic changes are correlated with the animal's tinnitus status.

## Introduction

Acoustic trauma is the most common cause of hearing loss and tinnitus in humans (Cooper, 1994). In fact, noise exposure is the most frequently reported cause of occupational disorders in the world (Nelson et al., 2005; Dobie, 2008) and the fourth leading cause of medical referral for combatants returning from deployment (Schulz, 2004). Therefore, the consequences of exposure to acoustic trauma will impose a significant negative economic burden on healthcare systems worldwide. Using tinnitus as an example, this phantom sound is severe enough in 1% of adults to affect their day-to-day normal life (Vio and Holme, 2005). In severe cases, it can be extremely disturbing, and even lead to suicide (Shargorodsky et al., 2010). In the USA, tinnitus affects 25% of the population at some stage in their life, with 8% of people experiencing persistent or chronic tinnitus (Shargorodsky et al., 2010), and this is similar to the prevalence for the rest of the world (Henry et al., 2005; McCormack et al., 2014; Wu et al., 2015). While the prevalence of tinnitus normally increases with age, noise exposure is believed to be the most common cause of tinnitus in humans (Cooper, 1994). The prevalence of tinnitus is increased by several-fold among military populations, especially those who have served in battle zones (Shah et al., 2014). A recent study analyzed the incidence of hearing loss and tinnitus in a group of people who work in a noisy environment and found that approximately 40% of them had tinnitus (Hong et al., 2016). It is also alarming that an increasing number of adolescents and young adults are experiencing tinnitus due to risky music-listening behaviors (Vogel et al., 2014).

Tinnitus treatment options are very limited and none of the currently available approaches, such as hearing aids, sound masking, drug treatments, hyperbaric oxygen therapy, acupuncture or neuromodulation, show benefit following meta-analytic scrutiny (Hesser et al., 2011). There is no FDA-approved drug marketed for tinnitus, but a wide variety of drugs have been prescribed off-label with no added benefits (Langguth and Elgoyhen, 2012). One of the reasons accounting for the lack of effective treatment for tinnitus is its heterogeneity. First, there is large etiologic heterogeneity in tinnitus. Tinnitus can be caused by many factors including noise exposure, head and neck injury, drug toxicity, ear infection, Meniere's disease, aging and even affective disorders, such as depression (see Baguley et al., 2013, for a review), which suggests that the underlying mechanisms of tinnitus can be very different for different causes. Second, heterogeneity also exists in an individual's reaction to tinnitus. For example, tinnitus severity or tinnitus-related distress is not always proportional to the loudness of the sound of the tinnitus, but can be related to certain personality traits such as anxiety, depression, sleeping difficulties and life satisfaction (Langenbach et al., 2005; Langguth et al., 2007). Therefore, it is not surprising that the outcomes of currently available tinnitus therapies are highly variable (Hoare et al., 2011).

Traditionally, the gold standard, modern drug discovery approach seeks to design more selective drugs with ideally one specific target in order to reduce adverse side effects. Despite excessive efforts and investment routinely made in discovering individual molecular targets over the last two decades, the rate of new drug candidates being translated to clinical use is decreasing (Kola and Landis, 2004). Hopkins (2008) argued that the “one gene, one drug, one disease” drug design philosophy might be the fundamental problem. This is because biological functions, or dysfunctions in disease status, are more likely to be a consequence of complex biochemical regulation processes driven by interactive networks within the genome (Chen et al., 2008), transcriptome (Iancu et al., 2014), proteome (Ebhardt et al., 2015), and metabolome (Shah et al., 2015). Targeting such dynamic network biology by identifying disease-causing networks rather than disease-causing genes, is likely to be a more effective approach for drug discovery (Roth et al., 2004; Hopkins, 2007, 2008; Kell and Goodacre, 2014).

Since genomics, epigenetics, transcriptomics, and proteomics all converge at the level of metabolomics and changes in metabolite concentrations are more substantial and defining than signals in other “omics,” metabolomics is considered to be the integration of all “omics.” Therefore, it is more reliable, sensitive and powerful in reflecting changes in biological functions due to either disease or drug action. By systematically analyzing low molecular weight metabolites in biological samples, metabolomics has been used to discover new biomarkers for diagnosis, monitoring and understanding the mechanisms of diseases and drugs in many areas, such as cancer (Olivares et al., 2015), diabetes (Li et al., 2013), psychotic disorders (Sethi and Brietzke, 2016), depression (Huang and Lin, 2015), cardiovascular diseases (Rankin et al., 2014), Alzheimer's disease (Graham et al., 2015), and epilepsy (Loeb, 2011).

In comparison, a significant gap in tinnitus research and drug discovery is readily apparent. On the one hand, numerous studies focus on one type of receptor, ion channel, neurotransmitter or neuron in one area of the brain or one particular pathway through molecular, electrophysiological and pharmacological studies seeking to elucidate the underlying neural mechanisms to cure tinnitus (Milbrandt et al., 2000; Liu et al., 2003; Eggermont and Roberts, 2004; Brozoski et al., 2007, 2012; Kaltenbach and Godfrey, 2008; Dong et al., 2010; Middleton et al., 2011; Zhang et al., 2011; Richardson et al., 2012; Zheng et al., 2012; Bauer et al., 2013; Kalappa et al., 2014). In contrast, advanced neuroimaging technology reveals tinnitus-associated changes in neuronal activity and connectivity involving multiple neural networks, both in human patients (Leaver et al., 2011; Vanneste et al., 2011; Kraus and Canlon, 2012; Maudoux et al., 2012; Song et al., 2012; Vanneste and De Ridder, 2012; Boyen et al., 2014; Husain and Schmidt, 2014) and an animal model (Chen et al., 2014). It has been increasingly recognized by the international tinnitus research community that tinnitus is unlikely to be generated by a single pathological source, but rather by complex network changes involving not only the auditory system but also systems related to memory, emotion and stress (see Roberts et al., 2010; Henry et al., 2014; Simonetti and Oiticica, 2015; Leaver et al., 2016, for reviews). One obvious and significant gap is a lack of biomarkers that reflect the consequences of this interactive “tinnitus-causing” network. So far, only one study has attempted to identify protein targets for tinnitus by analyzing the side effect targets for 275 drugs that cause tinnitus (Elgoyhen et al., 2014). Although this is the first study using a network approach to identify targets related to tinnitus, the significance is limited by the fact that tinnitus as a side effect may not necessarily be generated through the drug's pharmacological targets that were analyzed in the study. Therefore, a more direct approach by analyzing metabolite profiles in relation to tinnitus may provide insights into the phenotype, underlying pathophysiology as well as potential therapeutic targets of acoustic trauma and tinnitus. In the present study, we made the first attempt in analyzing metabolite profiles in different brain areas of rats at 6 months after exposure to acoustic trauma. This was a pilot study in which tissue from animals that had been subjected to acoustic trauma, but no longer exhibited tinnitus-related behavior, was used to investigate the potential application of metabolomics to the study of the effects of acoustic trauma on the brain, with a view to its potential relevance for tinnitus in the longer term.

## Methods

### Animals and tissue sample collection

The University of Otago Committee on Ethics in the Care and Use of Laboratory Animals approved all experimental procedures.

All the tissue samples were collected from our previous study which investigated the effects of L-baclofen on acoustic trauma-induced tinnitus (see Zheng et al., 2012, for details). Briefly, 16 male Wistar rats were divided into sham and acoustic trauma groups (*n* = 8 per group) and tested for the behavioral signs of tinnitus before receiving vehicle and 3 doses of L-baclofen (1, 3, and 5 mg/kg). Following the last L-baclofen treatment (5 mg/kg), a 2-week washout period was allowed before the final tinnitus behavioral testing (Zheng et al., 2012). At the end of the final tinnitus testing, the animals were sacrificed and the brains were rapidly removed, placed on ice and dissected into 12 different brain regions. These included the cochlear nucleus (CN), vestibular nucleus complex (VNC), inferior colliculus (IC), superior colliculus (SC), auditory cortex (AC), subregions of the hippocampus [CA1, CA2/3 and dentate gyrus (DG)], frontal cortex (FC), perirhinal cortex (PC), entorhinal cortex (ERC), and cerebellum (CB). Dissections were made according to the Paxinos and Watson brain atlas (Paxinos and Watson, 2008). Tissues were snap frozen on dry ice and kept at −80°C until the time of the assay.

### Acoustic trauma

Unilateral acoustic trauma was delivered using the procedure described in Zheng et al. (2012). Animals were anesthetized with ketamine HCl (75 mg/kg, s.c.) and medetomidine hydrochloride (0.3 mg/kg, s.c.) and then placed inside a sound attenuation chamber for a 1 h exposure to a 16 kHz 110 dB SPL pure tone delivered to one of the ears (Zheng et al., 2012). The pure tone was generated by an NI 4461 Dynamic Signal Acquisition and Generation system (National Instruments New Zealand Ltd) and was delivered to one of the ears through a closed field magnetic speaker with a tapered tip (Tucker-Davis Technologies), attached to a 3-mm cone-shaped speculum that was fitted tightly into the external auditory canal. The sound pressures were calibrated before noise exposure by connecting the speaker to a -inch prepolarized free-field microphone (Type 40BE, GRAS Sound, and Vibration) via the speculum used to fit into the external auditory canal. The unexposed ear was blocked with cone-shaped foam and taped against the foam surface. The sham animals were kept under anesthesia for the same duration as the noise trauma animals, but without noise exposure.

### Auditory brainstem-evoked potentials

The auditory function of both ears of the exposed and sham animals before and immediately after the acoustic trauma or sham treatment was measured using auditory brainstem-evoked response (ABR) thresholds, as described in Zheng et al. (2012). Briefly, the animals were anesthetized as described in the Section Acoustic Trauma and subdermal needle electrodes were placed at the vertex and over the bullae with a reference electrode at the occiput. ABR thresholds were tested for tone bursts (2 ms rise/decay, 1 ms plateau) presented at a rate of 50/s, in a decreasing intensity series, beginning with levels that elicited distinct evoked potentials. Hearing threshold was indicated by the lowest intensity that produced visually distinct potentials. Unilateral acoustic trauma produced an immediate elevation of the ABR threshold in the exposed ear across all of the frequencies tested (Zheng et al., 2012). However, the auditory function of the ears of sham exposed animals and the unexposed ears of the acoustic trauma exposed animals, were not affected. Although we did not test the animal's ABR thresholds at 6 months post-exposure prior to sacrificing the animals in this study, we reported in a separate study that ABR thresholds in the exposed ear returned to the same level as that in sham animals at 6 months after exposure (Zheng et al., 2015). This suggests that hearing loss caused by the specific acoustic trauma we used is not permanent and is most likely to subside by 6 months post-exposure.

### Tinnitus assessment

In the original study of Zheng et al. (2012), from which the tissue used in this study was taken, the presence of tinnitus was tested after the acoustic trauma using a conditioned lick suppression paradigm, as described in detail in Zheng et al. (2012). Briefly, tinnitus assessment was conducted in an operant conditioning test chamber in which drinking activity was measured by a lickometer with a photobeam. A speaker generated broadband noise (BBN; white noise ranging from 3 to 20 kHz) or pure tones of different frequencies and intensities via a sound generator. The chamber floor delivered an electric shock produced by a constant current shock source. The conditioned lick suppression paradigm consisted of 15 min of testing daily and the animals went through an acclimation phase, a Pavlovian conditioned suppression training phase, and a frequency discrimination phase. During the acclimation phase, the BBN was played throughout the 15 min session except at 10 random intervals, at which point 15 s acoustic stimuli were inserted. Two of the 10 presentations were always speaker off periods (i.e., silence) and the remaining 8 were one of BBN, 10 kHz tones or 20 kHz tones at one of 4 different intensity levels in a random order with each stimulus repeated twice within each session. The type of stimulus varied randomly between sessions, but remained constant within a session. Following acclimation, each animal received conditioned suppression training in which a 3 s foot shock (0.35 mA) was presented at the end of each speaker off period. The foot shock acted as an unconditioned stimulus (UCS) and the speaker off period acted as a conditioned stimulus (CS). Over a few sessions, the rats reacted to the speaker off by stopping licking (i.e., the conditioned suppression). Once the lick suppression was established, the rats were subjected to the frequency discrimination test, during which the acoustic stimuli were presented in the same way as in the acclimation and the suppression training. If a rat did not have tinnitus, the presentation of the stimuli had no effect on its drinking activity. However, if a rat had tinnitus, the tinnitus served as the conditioned stimulus (CS) during the training sessions, therefore, a stimulus with sensory features resembling tinnitus during the testing session would produce greater suppression. Tinnitus was assessed in these rats at 2 weeks after the noise exposure, during the L-baclofen or vehicle treatment as well as at the end of the experiment (Zheng et al., 2012).

### Tissue preparation

Frozen brain tissue samples were ground to an homogeneous powder using a liquid nitrogen-chilled mortar and pestle and rapidly transferred to a 1.5 mL microcentrifuge tube. Nine hundred μl of methanol containing the internal standard, [^13^C_2_]-myristic acid (12.5 μg/ml), was added to each tube. The mixture was vigorously vortexed for 3 min and centrifuged at 20,000 g for 10 min at 4°C.

An aliquot of 100 μl supernatant was transferred to a gas chromatography (GC) vial and evaporated to dryness using a SPD2010-230 SpeedVac Concentrator (Thermo Savant, Holbrook, USA). The dried residue was then dissolved in 30 μl of methoxyamine in pyridine (10 mg/ml) and vigorously vortexed for 2 min. The methoximation reaction was carried out for 16 h at room temperature, followed by trimethylsilylation for 1 h by adding 30 μl of *N*-methyl-*N*-(trimethylsilyl)trifluoroacetamide (MSTFA) with 1% trimethylchlorosilane (TMCS) as the catalyst. Finally, the solution was vortex-mixed again for 30 s after adding the external standard, methyl myristate in heptane (30 μg/ml), to each GC vial for GC/mass spectrometry (MS) analysis.

### GC/MS

Chromatographic separation of the analytes was achieved with a Shimadzu GCMSQP2010 (Shimadzu Corp., Tokyo, Japan) equipped with a RTx-5MS column (30 mm × 0.25 mm i.d. fused-silica capillary column chemically bonded with a 0.25 μm cross bond, 5% diphenyl/95% dimethyl polysiloxane, Restek Corporation, PA, USA). Helium was used as the carrier gas and the temperature was initially set at 80°C for 3 min, which was then increased to 300°C at 20°C/min. Once the temperature was at 300°C, it was maintained for another 3 min. The eluate was introduced through the transfer line into the mass spectrometer, where the molecules were ionized with a current beam of 70 eV. The masses were scanned over m/z 50–700 with a detector voltage of −1050 V. To minimize systematic variations, all samples were analyzed in a randomized order, and the quantitative data were normalized to the internal standard. The metabolites were identified by automatically comparing the MS spectra, in-source fragments and ion features of each peak in the experimental samples with those of reference standards or those available in libraries, such as mainlib and publib in the National Institute of Standards and Technology (NIST) library 2.0 (2012); Wiley 9 (Wiley-VCH Verlag GmbH & Co. KGaA, Weinheim, Germany); the in-house mass spectra library database established by Umeå Plant Science Center (Umeå University, Sweden) and our own Laboratory at China Pharmaceutical University.

### Data analysis

Mean differences between the sham and acoustic trauma groups were compared using two-sample independent Student's *t*-tests. However, since the metabolomics data have multiple dependent variables, it required multivariate statistical analysis that takes account of changes in systems of variables and would be sensitive to changes that may occur at the system level between sham and acoustic trauma animals. One approach to analyzing changes in systems of variables is principal component analysis (PCA), in which large numbers of variables (i.e., “high dimensional data”) are reduced to components or eigenvalues that represent combinations of variables that account for most of the variation in the data (Manly, 2005). In PCA, these components also have weightings or “eigenvectors” that indicate the weight of each variable within a component. The different components are intended to be independent of one another (“orthogonal”) so that they capture unique aspects of the variation in the data. PCA, unlike Factor Analysis, does not assume a formal statistical model and therefore assumptions such as multivariate normality are unnecessary. PCA was performed on the correlation matrix (i.e., the data were z transformed) (Manly, 2005). Loading plots were used to determine the clustering of variables and whether their relationship within components changed as a result of acoustic trauma. Another way of analyzing multivariate changes is linear discriminant analysis (LDA), which attempts to identify a linear equation that combines the metabolomics variables, with different weightings, in order to predict whether animals belong to the sham or acoustic trauma groups. Partial least squares discriminant analysis (PLS-DA, equivalent to “projection to latent structures DA”) is a method that uses partial least squares regression in the discriminant analysis, which can be used to elucidate the separation between groups of variables through rotating the principal components obtained in PCA, and is particularly useful when there are more variables than observations and when there is correlation or “multicollinearity” amongst the variables (Tang et al., 2014). In orthogonal projection to latent structures DA (OPLS-DA), an extension of PLS-DA, the data from the continuous variables in the discriminant analysis function are separated into those that are predictive of the dependent variable (e.g., sham or acoustic trauma) and those that are not, resulting in enhanced diagnostics. We performed OPLS-DA and then tested the efficacy of the discriminant functions in predicting whether the animals were exposed to acoustic trauma, using cross-validation (Manly, 2005). The goodness of fit for a model was evaluated using three quantitative parameters: i.e., R^2^X, the explained variation in X, R^2^Y, the explained variation in Y, and Q^2^Y, the predicted variation in Y based on the model using cross-validation (Sedghipour and Homayoun Sadeghi-Bazargani, 2012). The range of these parameters is between 0 and 1; the closer they approach 1, the better they can be predicted or explained. All analyses were carried out using R (R Core Team, 2013). In formal statistical tests, *P* ≤ 0.05 was considered significant. Variable importance was used to summarize the importance of the X-variables, both for the X- and Y-models and was measured by the variable influence on projection (VIP). The VIP value is a parameter indicating the importance of a variable that contributes to the model. In a model, one can compare the VIP of one variable to the others. Variables with a large VIP, larger than 1, are the most relevant for explaining Y. The S-plot is an easy way to visualize an OPLS discriminant analysis model of two classes, since it provides visualization of the OPLS/OPLS-DA predictive component loading to facilitate model interpretation (Wiklund et al., 2008). The S-plot is used to visualize both the covariance and the correlation structure between the X-variables and the predictive score t[1]. Thus, the S-plot is a scatter plot of the p[1] vs. p(corr)[1] vectors of the predictive component. The axes in the S-plot from the predictive component are p1 and p(corr)1, representing the magnitude (modeled covariation) and reliability (modeled correlation), respectively. Hence, in the S-plot both magnitude (intensity) and reliability are visualized. In spectroscopic data, the magnitude of the peak is very important as peaks with low magnitude are close to the noise level and thus have a higher risk for spurious correlation. Ideally, biomarkers would have both high reliability and magnitude. This plot often takes the shape of the letter “S.” X-variables situated far out on the wings of the S shape represent higher model influence with higher reliability and are of relevance in the search for biomarkers that are up- or down-regulated. In addition, metabolic pathway analysis was performed by inputting discriminant molecules into Metaboanalyst (available online at http://www.metaboanalyst.ca) and network analysis was processed by Metscape 3.1-based on Cytoscape 3.3.0. “Relative abundance” was calculated by summing the integral areas of metabolites with high variable importance scores, normalizing them, and comparing the sham vs. acoustic trauma groups using a two-sample, independent Student's *t*-test (Gray et al., 2015).

## Results

**Figure 1:**
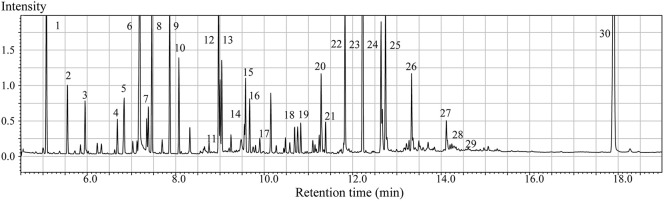
**Typical GC/MS chromatograms of extracts from brain tissue of a sham animal**. The compounds were identified as: 1. Lactic Acid 2. Alanine 3. Oxalic acid 4. Valine 5. Urea 6. Phosphoric acid 7. Proline 8. Glycine 9. Serine 10. Threonine 11. Malic acid 12. Aspartic acid 13.γ-Aminobutanoic acid 14.Creatinine 15. Glutamic acid 16. Phenylalanine 17. N-Acetylaspartic acid 18. Hypoxanthine 19. Citric acid 20. Lysine 21. Tyrosine 22. Palmitic acid 23. Myo-Inositol 24. Oleic acid 25. Stearic acid 26. Arachidonic acid 27. Docosahexaenoic acid 28. Inosine 29. Glycerol monostearate 30. Cholesterol

GC/MS analysis of the brain tissue extracts revealed a large number of peaks (Figure 1). Deconvolution of the chromatograms produced a total of 107 distinct peaks and 88 were authentically identified by comparing the mass spectrum of the peak with that available in the libraries and that of the reference compounds. These included amino acids, small organic acids, carbohydrates, fatty acids, lipids, and amines. To acquire the quantitative data, a feature mass (m/z) was chosen for each peak, and the peak area was obtained for each deconvoluted peak/molecule.

**Figure 2:**
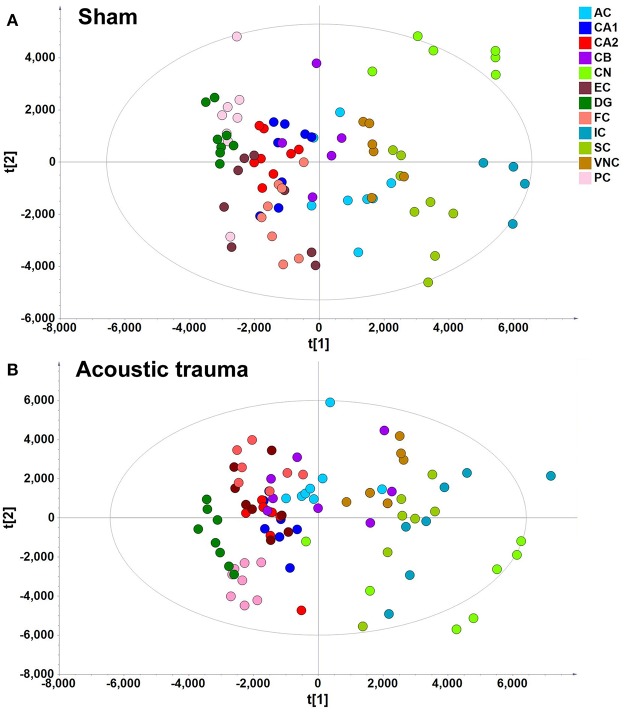
**PLS-DA score plot of different brain regions from sham (A)** and acoustic trauma **(B)** groups. **(A)** sham (PC1: R^2^X = 0.344, R^2^Y = 0.0842, Q2 = 0.0726; PC2: R^2^X = 0.249, R^2^Y = 0.0525, Q2 = 0.0373; All 6 PCs: R^2^X (cum) = 0.84, R^2^Y (cum) = 0.381, Q2 (cum) = 0.286.); **(B)** acoustic trauma (PC1: R^2^X = 0.336, R^2^Y = 0.0759, Q2 = 0.0683; PC2: R^2^X = 0.307, R^2^Y = 0.0513, Q2 = 0.0407; All 6 PCs: R^2^X (cum) = 0.875, R^2^Y (cum) = 0. 384, Q2 (cum) = 0.325. AC, auditory cortex; CB, cerebellum; IC, inferior colliculus; CN, cochlear nucleus; VCN, vestibular nucleus complex; SC, superior colliculus; CA1 and CA2 of the hippocampus; DG, dentate gyrus; FC, frontal cortex; PC, perirhinal cortex; EC, entorhinal cortex.

A data matrix of molecules in samples from the sham and acoustic trauma animals for the 12 brain areas was initially analyzed using PCA and PLS-DA. PLS-DA score plots, in which the scores for PC2 (i.e., t[2]) were plotted against those for PC1 (t[1]), were used to show the distribution of the analyzed samples containing the information from all the metabolites in different brain areas (Figure 2). According to the PLS-DA algorithm, each dot represents the summarized information from all the 88 molecules measured in a single sample for a particular brain region. Therefore, the distance between the dots indicates the similarity of the metabolic composition between the samples, i.e., the closer they cluster together the more similar they are. An overview of the PLS-DA score plot of the sham animals revealed that the composition of metabolites varied between different brain regions; however, brain regions with similar functions seemed to have a similar metabolite composition (Figure 2A). For example, metabolites from the temporal lobe area, such as the CA1, CA2/3, DG, ERC, and PC, were located close to each other on the PLS-DA score plots but were further apart from those from midbrain and hindbrain areas. Animals that received acoustic trauma showed similar metabolite clustering to the sham animals (Figure 2B). This was confirmed by an OPLS-DA analysis, which generated a model comparing the total metabolites in all the brain areas between sham and acoustic trauma animals (PC1: R^2^X = 0.21, R^2^Y = 0.0286, Q^2^Y = −0.0245; PC2: R^2^X = 0.112, R^2^Y = 0.0354, Q^2^Y = −0.038). OPLS-DA visualized the clustering samples within a group and the separation of two groups of samples. The closer clustering of samples indicates the more similar composition of the detected variables, while the more distant scattering of samples indicates the larger variation in the composition of the detected variables. These results suggested that, taking all brain regions together, the acoustic trauma and sham animals could not be discriminated from one another.

**Figure 3:**
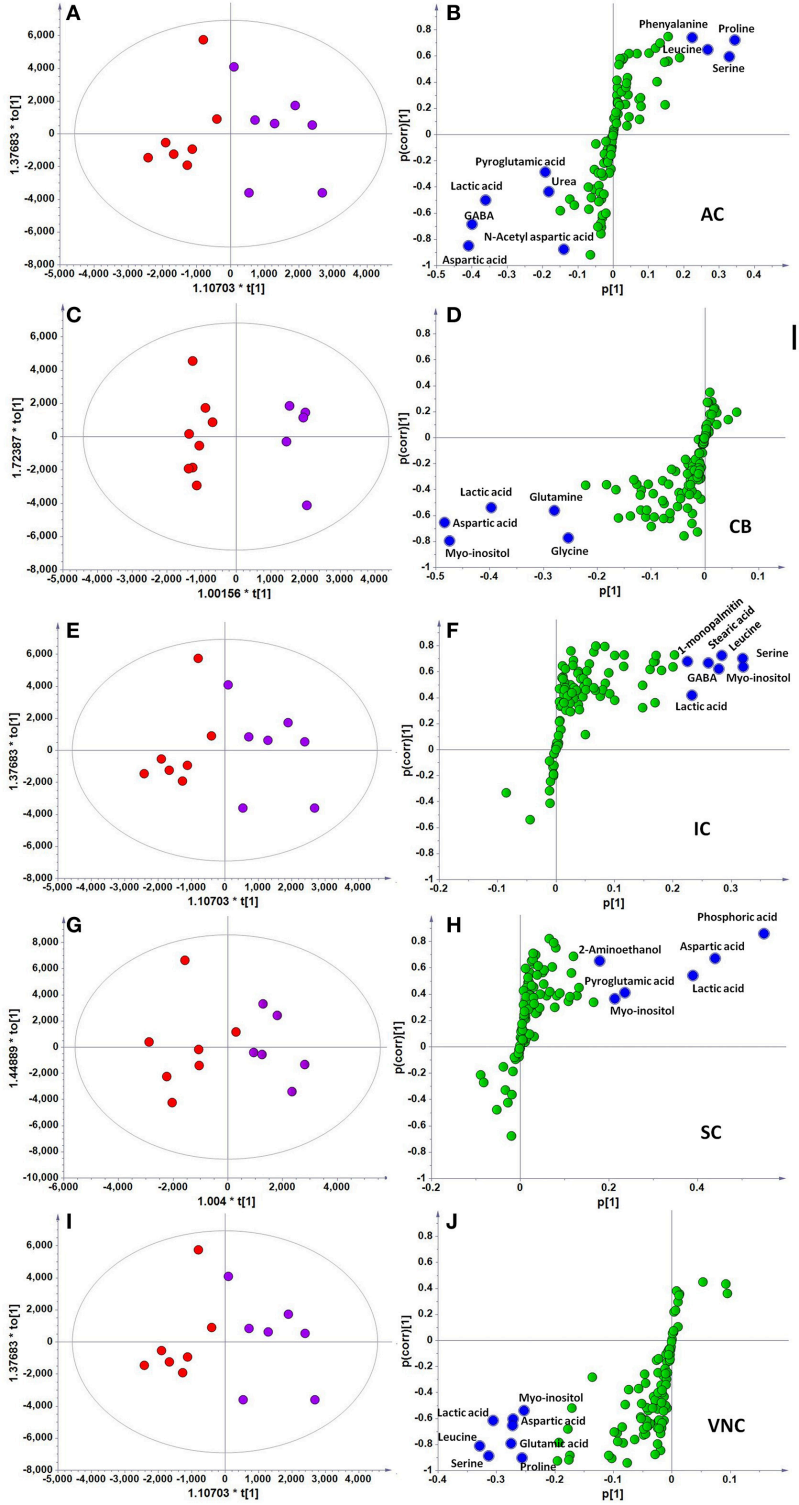
**OPLS-DA and S-plot analysis comparing the OPLSDA scores between sham and acoustic trauma animals in different brain regions**. Left panel, OPLSDA scores plots, red dots: Sham, purple dots: Acoustic trauma; Right panel, S-plots. **(A,B)** AC (Predictive component: R^2^X = 0.194, R^2^Y = 0.76, Q2 = 0.45; Orthogonal component 1: R^2^X = 0.446; All components: R^2^X (cum) = 0.64); **(C,D)** CB (Predictive component: R^2^X = 0.152, R^2^Y = 0.973, Q2 = 0.68; Orthogonal component 1: R^2^X = 0.364; All components: R^2^X (cum) = 0.927); **(E,F)** IC (Predictive component: R^2^X = 0.293, R^2^Y = 0.978, Q2 = 0.702; Orthogonal component 1: R^2^X = 0.417; All components: R^2^X (cum) = 0.905); **(G,H)** SC (Predictive component: R^2^X = 0.238, R^2^Y = 0.791, Q2 = 0.691; Orthogonal component: R^2^X = 0.562; All components: R^2^X (cum) = 0.8); **(I,J)** VNC(Predictive component: R^2^X = 0.403, R^2^Y = 0.779, Q2 = 0.445; Orthogonal component: R^2^X = 0.389; All components: R^2^X (cum) = 0.792). AC, auditory cortex; CB, cerebellum; IC, inferior colliculus; CN, cochlear nucleus; VCN, vestibular nucleus complex.

**Figure 4:**
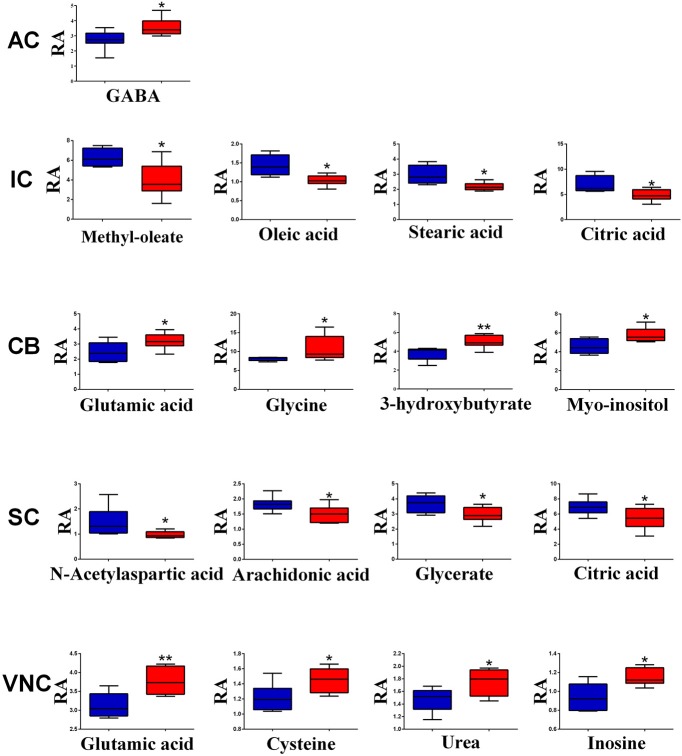
**Relative abundance (RA) of the discriminant metabolites in different brain regions of sham (blue) and acoustic trauma (red) groups**. ^*^p < 0.05; ^**^p < 0.01. AC, auditory cortex; CB, cerebellum; IC, inferior colliculus; CN, cochlear nucleus; VCN, vestibular nucleus complex.

However, when the metabolite profiles in individual areas were analyzed using the OPLS-DA model, acoustic trauma (purple dots in left panel, Figure 3) significantly shifted the metabolite profile away from the sham animals (red dots in right panel, Figure 3) in the AC (Predictive component: R^2^X = 0.194, R^2^Y = 0.76, Q^2^Y = 0.45; Orthogonal component: R^2^X = 0.446; All components: R^2^X(cum) = 0.64), IC (Predictive component: R^2^X = 0.293, R^2^Y = 0.978, Q^2^Y = 0.702; Orthogonal component 1: R^2^X = 0.417; All components: R^2^X (cum) = 0.905), SC (Predictive component: R^2^X = 0.238, R^2^Y = 0.791, Q^2^Y = 0.691; Orthogonal component: R^2^X = 0.562; All components: R^2^X (cum) = 0.8), VNC (Predictive component: R^2^X = 0.403, R^2^Y = 0.779, Q^2^Y = 0.445; Orthogonal component: R^2^X = 0.389; All components: R^2^X (cum) = 0.792) and CB (Predictive component: R^2^X = 0.152, R^2^Y = 0.973, Q^2^Y = 0.68; Orthogonal component 1: R^2^X = 0.364; All components: R^2^X (cum) = 0.927) (Figure 3, left panel). In addition, the discriminant molecules or potential markers for the two groups could be visualized in an S-plot (Figure 3, right panel). The X- and Y-axes are p[1] and p(corr)[1] vectors of the predictive component, which describes the magnitude and reliability of each variable in X, respectively. X-variables situated far out on the tips of the wings of the S shape represent high model influence with high reliability and are of relevance in the search for biomarkers. Further analysis of the relative concentrations using Student's *t*-tests revealed a total of 17 differentially changed molecules associated with acoustic trauma (Figure 4). These included urea, amino acids, fatty acids, sugar acids, nucleosides and organic acids and the changes were region-specific. For example, the level of GABA was significantly increased only in the AC, while an increase in glutamic acid was observed in both the CB and VNC (Figure 4).

**Figure 5:**
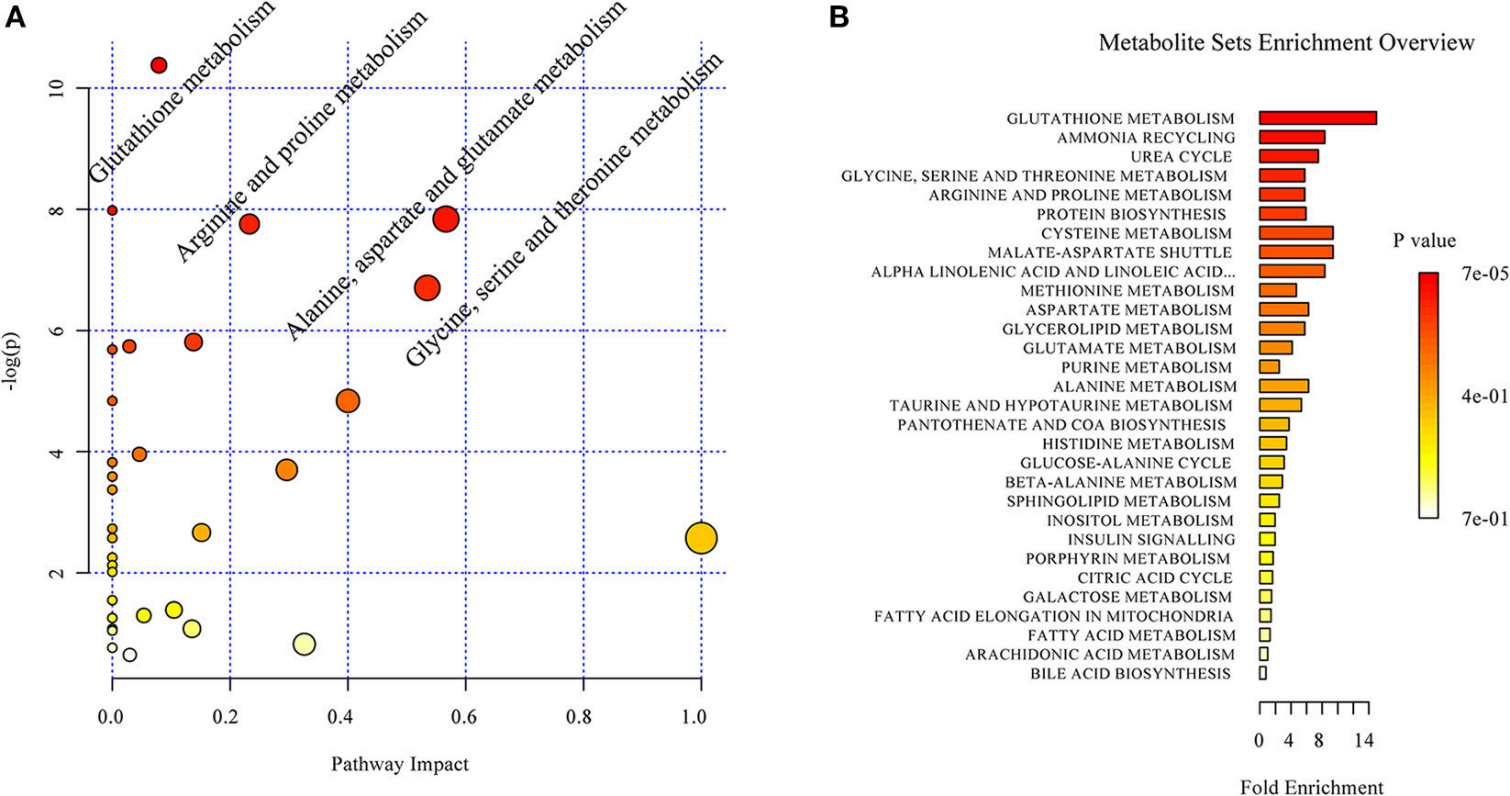
**Overview of the impact of acoustic trauma on brain metabolites. (A)** The pathway impact of acoustic trauma on metabolites. The y axis shows the *p*-values and the x axis, the pathway impact values; the node color is based on its *p*-value and the node size reflects the pathway impact values. **(B)** The enrichment overview of the pathway-associated metabolite sets perturbed by acoustic trauma.

Based on the discriminant molecules identified, a metabolic pathway analysis was conducted by entering the discriminant molecules into the available online Metaboanalyst programme. The pathway impact analysis suggested that acoustic trauma produced a significant impact on a number of metabolic pathways. A set of 4 pathways was selected. These included glutathione metabolism, alanine aspartate and glutamate metabolism, arginine and proline metabolism and glycine, serine and threonine metabolism (Figure 5A) The enrichment overview of the network ranked the acoustic trauma-perturbed metabolite sets by their respective fold enrichment, which provides information on the significant and coordinated changes in metabolites. As shown in Figure 5B, the identified metabolites were involved in multiple pathways and the *p*-values indicated whether a particular metabolite set was represented more than expected by chance. The most enriched pathway was the glutathione metabolism pathway.

**Figure 6:**
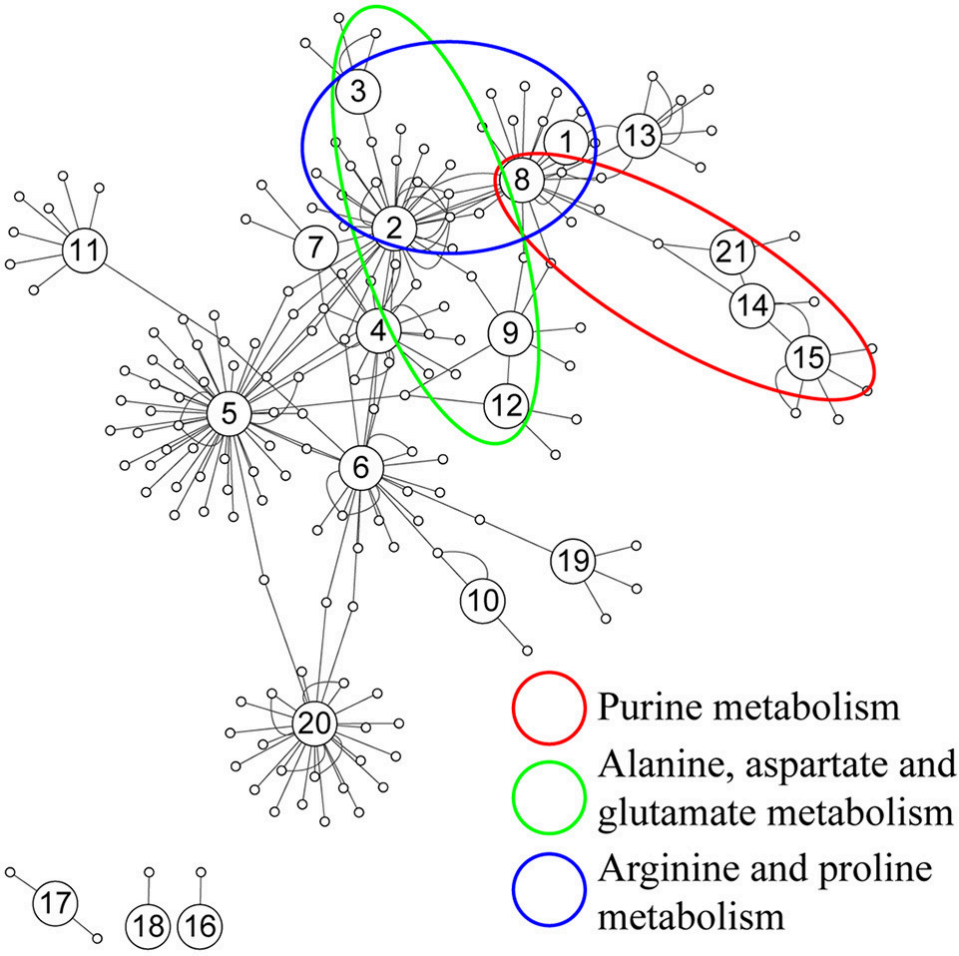
**Correlation network analysis of differential metabolites**. (1) N-Acetylaspartate, (2) Glutamic acid, (3) γ-aminobutyric acid, (4) Cysteine, (5) Glycine, (6) Serine, (7) Pyroglutamic acid, (8) Aspartic acid, (9) Ornithine, (10) Glyceric acid, (11) Myo-Inositol, (12) Urea, (13) Citric acid, (14) Hypoxanthine, (15) Xanthine, (16) 3-Hydroxybutyric acid, (17) Oleic acid, (18) Stearic acid, (19) Palmitic acid, (20) Arachidonic acid, (21) Inosine.

Further analysis on the correlation was conducted based on the 88 identified metabolites from both sham and acoustic trauma animals to further study the association and interaction patterns between metabolites. Nodes represent the identified metabolites and edges indicate significant correlation between nodes. As can be seen in Figure 6, some nodes had more connections with others, while other nodes had fewer links. There were also nodes that were clustered together with a short distance between them. Based on the degree (k) of each node, the top 10 highly connected metabolites were considered as hubs of the network and these metabolites are involved in purine metabolism (green circle), glutamate metabolism (blue circle) and arginine metabolism (red circle) (Figure 6).

## Discussion

We reported here that acoustic trauma in rats caused a significant shift in metabolite profiles in a number of brain areas. These included both auditory and non-auditory areas. In addition, acoustic trauma imposed a significant impact on a number of metabolic pathways including those involved in purine, glutamate and arginine metabolism.

To our knowledge, this is the first study that has used metabolomics to profile brain metabolite changes in relation to acoustic trauma. Over the last decade, metabolomics has become one of the major “omics” tools in understanding disease pathology, identifying biomarkers, improving diagnosis and developing personalized therapy (see Botas et al., 2015; Shah et al., 2015 for reviews). In addition, brain metabolomics has been used to understand the pathology and identify potential markers for neurodegenerative diseases in both animal models and human post-mortem tissues due to the fact that metabolic changes in the brain are more likely to reflect disease etiology than those in peripheral biofluids (Pears et al., 2005; Salek et al., 2010; Graham et al., 2013; Fauvelle et al., 2015; Xu et al., 2016). In the present study, 107 small molecules were detected from 12 different brain regions and 88 of them were authentically identified. The metabolic models developed were able to accurately distinguish acoustic trauma animals from sham controls in selected brain regions. The predictive accuracies of these models ranged from 45 to 70.2%, which suggests a reasonably good level of predictive power when compared with 41% accuracy in predicting Batten's disease (Pears et al., 2005) and 54–60% in predicting Alzheimer's disease using animal brain tissues (Salek et al., 2010), although not as good as that reported using human post-mortem brain tissues for Alzheimer's disease (91–97%) (Graham et al., 2013).

Although it has neither been extensively studied and nor is well understood, hearing loss and/or tinnitus has been linked to changes in the homeostasis of body metabolism. For example, it has long been noticed that chronic kidney disease (Ikeda et al., 1987; Govender et al., 2013; Renda et al., 2015) or diabetes (Kurien et al., 1989; Sasso et al., 1999) that cause metabolic disturbances are often accompanied by impaired hearing. Moreover, metabolite changes have been associated with presbycusis (Profant et al., 2013), congenital sensorineural hearing loss (Wu et al., 2016) and idiopathic sudden sensorineural hearing loss (Dinc et al., 2016). In the present study, among the 88 small molecules identified, 17 of them had their levels significantly altered by acoustic trauma in at least one of the brain regions examined. These metabolites are predominantly involved in amino acid metabolism, the urea cycle as well as oxidation-reduction reactions. The long-term effects of acoustic trauma on amino acid levels in auditory brain regions have been investigated in a previous study, which reported a significant increase in glutamate, GABA and aspartate and a decrease in taurine (Godfrey et al., 2012). This is in a general agreement with the present results except that our study extended the finding into some non-auditory areas, such as the CB and VNC, which may suggest a more widespread effect of acoustic trauma in disturbing the balance between excitatory and inhibitory amino acids. It is of note that Bauer et al. (2013) have recently reported a link between the paraflocculus and tinnitus perception in rats. Using the network impact analysis, glutathione metabolism, the ammonia cycle and the urea cycle appeared to be affected the most by the acoustic trauma. While glutathione is an important antioxidant in cells, ammonia and urea are crucial to amino acid synthesis and metabolism. Studies have shown that a low protein diet resulting in a reduced cochlear glutathione level increased cisplatin-induced ototoxicity (Lautermann et al., 1995) and N-acetylcysteine (NAC), a precursor of glutathione, attenuated hearing loss in a number of animal models (Wu et al., 2010; Ding et al., 2016). Urea tests have long been used to aid the diagnosis of vestibular and hearing impairment in patients with Ménière's disease (Babin and Bumsted, 1980; Imoto and Stahle, 1983) and urea has also been used to temporarily improve hearing in these patients (Angelborg et al., 1977). This effect was thought to be partially due to the changes in perilymph osmolality caused by urea (Juhn et al., 1979). Interestingly, the urea cycle was altered in multiple brain regions in neurodegenerative diseases such as Huntington's disease (Patassini et al., 2015) and Alzheimer's disease (Xu et al., 2016), which suggests that brain urea metabolism may play an important role in maintaining neuronal function. Therefore, the altered brain glutathione and urea metabolism observed in the present study may contribute to acoustic trauma-induced hearing loss. It is worth mentioning that although hearing levels were not measured prior to sacrificing the animals in this study, we found in a separate study that hearing loss had recovered at 6 months following the same acoustic trauma exposure in rats (Zheng et al., 2015). Therefore, it is conceivable that hearing loss had also recovered by 6 months in the present study, hence, the metabolic changes were not directly attributable to hearing loss. It has been shown that acoustic trauma has variable effects on ABR thresholds in individual animals and the degree of hearing loss is not correlated with tinnitus perception in these mice (Longenecker et al., 2014; Longenecker and Galazyuk, 2016), which suggests that the impact of acoustic trauma on the auditory system is not limited to the peripheral damage, but involves damage to the higher centers of the brain. Our results are in agreement with this view and extend the evidence to include changes in metabolic pathways. In addition, the present study investigated metabolic changes in both auditory and non-auditory brain regions, which may provide insights into the wide range of emotional and cognitive impairments associated with acoustic trauma.

One of the limitations that prevented the current results being directly linked to tinnitus is the lack of behavioral evidence of tinnitus in these animals at the time of sample collection. As described in our previous publication (Zheng et al., 2012), the acoustic trauma-exposed animals developed tinnitus at 1 month post-exposure, which was confirmed by the conditioned lick suppression test. Both sham and exposed animals then received saline and 3 different doses of L-baclofen treatment and were tested for their behavioral signs of tinnitus after each treatment. At the conclusion of the experiment, the exposed animals did not have significant behavioral signs of tinnitus compared with sham animals. Therefore, it is not possible to relate the metabolic changes to tinnitus in this study, only to acoustic trauma. However, it is reasonable to attribute the changes to acoustic trauma since both the sham and acoustic trauma animals received exactly the same L-baclofen treatment. Nonetheless, this is the first study using the powerful metabolomics technique to investigate brain metabolite shifts in relation to acoustic trauma at the network level. This is a proof of concept study that established a method sensitive enough to detect metabolic changes in discrete brain regions.

## Conclusions

In this study, we optimized a metabolomics approach to predict acoustic trauma using brain tissues, with comparable accuracy to studies predicting other neurodegenerative diseases in animal models (Pears et al., 2005; Salek et al., 2010). Further studies are needed to identify brain metabolic changes specifically related to tinnitus and to correlate changes in the brain with those in the blood for future clinical translation. This will provide a powerful tool toward a better understanding of tinnitus heterogeneity and the development of personalized therapies.

## Ethics statement

All experiments were carried out in accordance with the New Zealand Animal Welfare Act 1999 and the University of Otago Code of Ethical Conduct for the Use of Animals. Formal approval to conduct the experiments described has been obtained from the University of Otago Committee for the Care and Use of Laboratory Animals.

## Author contributions

YZheng Corresponding author, secured the funding in New Zealand, designed the experiment, supervised the acoustic trauma model, collected the brain tissues, coordinated the collaboration, played a key role in the results interpretation and manuscript writing. GW 2nd corresponding author, secured the funding in China, contributed to the results interpretation and manuscript writing. DD Contributed to the results interpretation and manuscript writing. PS Secured the funding in New Zealand, contributed to the results interpretation and manuscript writing. JA Supervised the metabolomics experiments, analyzed the results and contributed to the results interpretation and manuscript writing. YZhu Performed the metabolomics experiments and contributed to data analysis. JH Performed the metabolomics experiments and contributed to data analysis.

### Conflict of interest statement

The authors declare that the research was conducted in the absence of any commercial or financial relationships that could be construed as a potential conflict of interest.
