# Corrigendum: Brain Metabolic Changes in Rats following Acoustic Trauma

**DOI:** 10.3389/fnins.2017.00260

**Published:** 2017-05-08

**Authors:** Jun He, Yejin Zhu, Jiye Aa, Paul F. Smith, Dirk De Ridder, Guangji Wang, Yiwen Zheng

**Affiliations:** ^1^Key Laboratory of Drug Metabolism and Pharmacokinetics, China Pharmaceutical UniversityNanjing, Jiangsu, China; ^2^Department of Pharmacology and Toxicology, School of Biomedical Sciences, University of OtagoDunedin, New Zealand; ^3^Brain Health Research Centre, University of OtagoDunedin, New Zealand; ^4^Brain Research New ZealandDunedin, New Zealand; ^5^Eisdell Moore Centre for Hearing and Balance Research, University of AucklandAuckland, New Zealand; ^6^Department of Neurosurgery, Dunedin Medical School, University of OtagoOtago, New Zealand

**Keywords:** metabolomics, acoustic trauma, tinnitus, brain, rats

In the original article, the same graph was used for Figures 3A, 3E and 3I by mistake. The corrected Figure 3 appears below.

The authors apologize for this error and state that this does not change the scientific conclusions of the article in any way.

**Figure 3:**
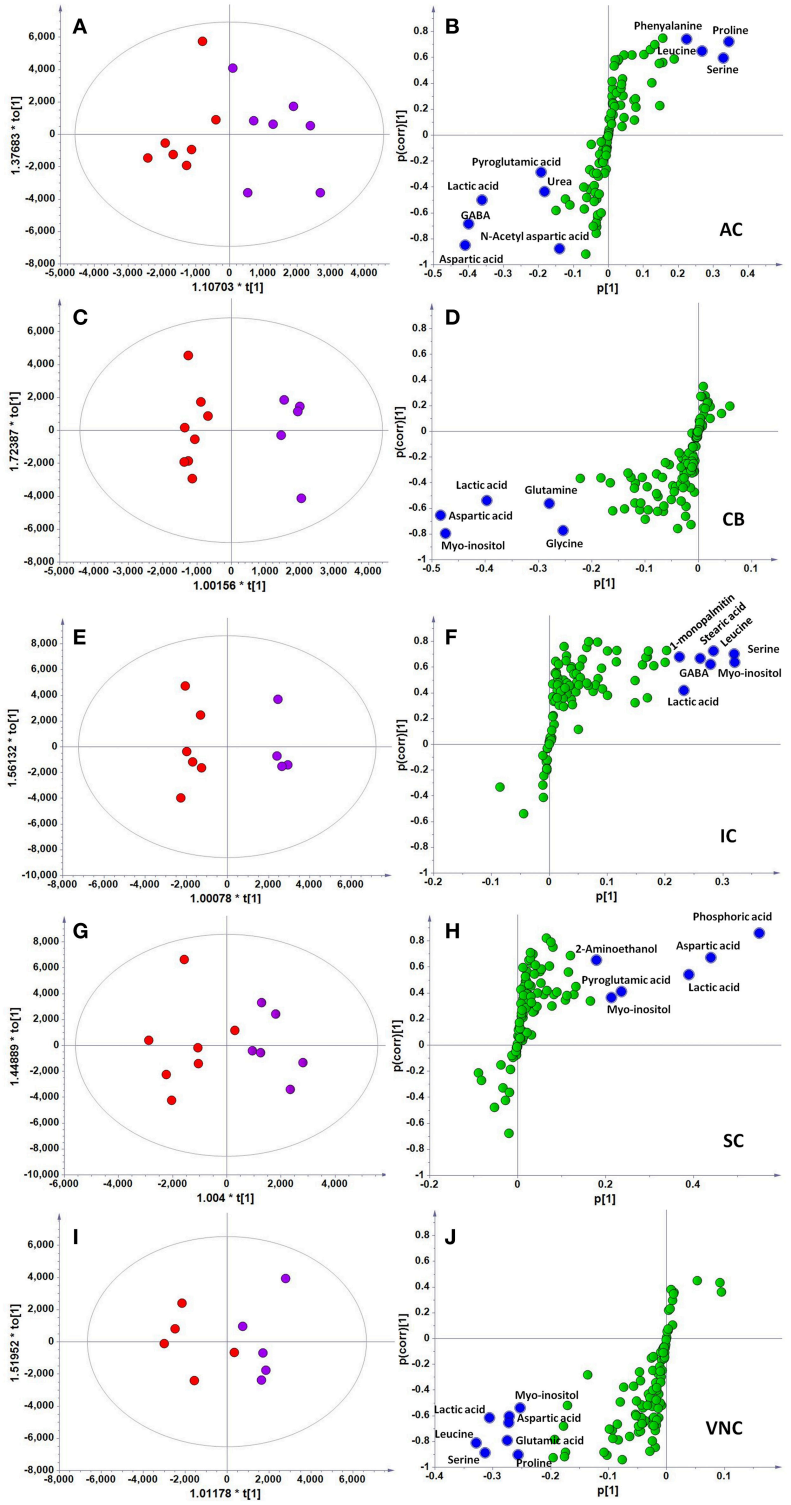
**OPLS-DA and S-plot analysis comparing the OPLSDA scores between sham and acoustic trauma animals in different brain regions**. Left panel, OPLSDA scores plots, red dots: Sham, purple dots: Acoustic trauma; Right panel, S-plots. **(A,B)** AC (Predictive component: R^2^X = 0.194, R^2^Y = 0.76, Q2 = 0.45; Orthogonal component 1: R^2^X = 0.446; All components: R^2^X (cum) = 0.64); **(C,D)** CB (Predictive component: R^2^X = 0.152, R^2^Y = 0.973, Q2 = 0.68; Orthogonal component 1: R^2^X = 0.364; All components: R^2^X (cum) = 0.927); **(E,F)** IC (Predictive component: R^2^X = 0.293, R^2^Y = 0.978, Q2 = 0.702; Orthogonal component 1: R^2^X = 0.417; All components: R^2^X (cum) = 0.905); **(G,H)** SC (Predictive component: R^2^X = 0.238, R^2^Y = 0.791, Q2 = 0.691; Orthogonal component: R^2^X = 0.562; All components: R^2^X (cum) = 0.8); **(I,J)** VNC(Predictive component: R^2^X = 0.403, R^2^Y = 0.779, Q2 = 0.445; Orthogonal component: R^2^X = 0.389; All components: R^2^X (cum) = 0.792). AC, auditory cortex; CB, cerebellum; IC, inferior colliculus; CN, cochlear nucleus; VCN, vestibular nucleus complex.

## Conflict of interest statement

The authors declare that the research was conducted in the absence of any commercial or financial relationships that could be construed as a potential conflict of interest.

